# Diabetes Self-Management Education Programs in Nonmetropolitan Counties — United States, 2016

**DOI:** 10.15585/mmwr.ss6610a1

**Published:** 2017-04-28

**Authors:** Stephanie A. Rutledge, Svetlana Masalovich, Rachel J. Blacher, Magon M. Saunders

**Affiliations:** 1 National Center for Chronic Disease Prevention and Health Promotion, CDC, Atlanta, Georgia; 2 Northrop Grumman, Atlanta, Georgia

## Abstract

**Problem/Condition:**

Diabetes self-management education (DSME) is a clinical practice intended to improve preventive practices and behaviors with a focus on decision-making, problem-solving, and self-care. The distribution and correlates of established DSME programs in nonmetropolitan counties across the United States have not been previously described, nor have the characteristics of the nonmetropolitan counties with DSME programs.

**Reporting Period:**

July 2016.

**Description of Systems:**

DSME programs recognized by the American Diabetes Association or accredited by the American Association of Diabetes Educators (i.e., active programs) as of July 2016 were shared with CDC by both organizations**.** The U.S. Census Bureau’s census geocoder was used to identify the county of each DSME program site using documented addresses. County characteristic data originated from the U.S. Census Bureau, compiled by the U.S. Department of Agriculture’s Economic Research Service into the *2013 Atlas of Rural and Small-Town America* data set. County levels of diagnosed diabetes prevalence and incidence, as well as the number of persons with diagnosed diabetes, were previously estimated by CDC. This report defined nonmetropolitan counties using the rural-urban continuum code from the *2013 Atlas of Rural and Small-Town America* data set. This code included six nonmetropolitan categories of 1,976 urban and rural counties (62% of counties) adjacent to and nonadjacent to metropolitan counties.

**Results:**

In 2016, a total of 1,065 DSME programs were located in 38% of the 1,976 nonmetropolitan counties; 62% of nonmetropolitan counties did not have a DSME program. The total number of DSME programs for nonmetropolitan counties with at least one DSME program ranged from 1 to 8, with an average of 1.4 programs. After adjusting for county-level characteristics, the odds of a nonmetropolitan county having at least one DSME program increased as the percentage insured increased (adjusted odds ratio [AOR] = 1.10, 95% confidence interval [CI] = 1.08–1.13), the percentage with a high school education or less decreased (AOR = 1.06, 95% CI = 1.04–1.07), the unemployment rate decreased (AOR = 1.19, 95% CI = 1.11–1.23), and the natural logarithm of the number of persons with diabetes increased (AOR = 3.63, 95% CI = 3.15–4.19).

**Interpretation:**

In 2016, there were few DMSE programs in nonmetropolitan, socially disadvantaged counties in the United States. The number of persons with diabetes, percentage insured, percentage with a high school education or less, and the percentage unemployed were significantly associated with whether a DSME program was located in a nonmetropolitan county.

**Public Health Action:**

Monitoring the distribution of DSME programs at the county level provides insight needed to strategically address rural disparities in diabetes care and outcomes. These findings provide information needed to assess lack of availability of DSME programs and to explore evidence-based strategies and innovative technologies to deliver DSME programs in underserved rural communities.

## Introduction

An estimated 29.1 million persons in the United States had diabetes in 2012, and this number is projected to reach 64 million by 2050 (*1*,*2*). Persons with diabetes have an increased risk for microvascular and macrovascular complications (e.g., heart disease, stroke, kidney disease, and retinopathy) that lead to a decrease in quality of life (*1*). The appropriate use of diabetes preventive care practices and adherence to self-management behaviors, such as routine medical visits, blood glucose and lipid tests, glucose self-monitoring, foot and eye examinations, and healthy dietary and physical activity, can prevent or delay costly complications (*3*).

Diabetes self-management education (DSME) is a clinical practice intended to improve preventive practices and behaviors with a focus on decision-making, problem-solving, and self-care (*4*). DSME increases the use of preventive care services and reduces glucose levels associated with diabetes complications in persons with diabetes (*4*). The National Standards for Diabetes Self-Management Education and Support define quality standards for DSME to support evidence-based care by diabetes educators (*5*). Ideally, DSME interventions should occur at four critical points (i.e., diagnosis, annual examinations, when complications arise, and with a change in care) and consider age, culture, and other factors (*4*).

Persons with a diagnosis of diabetes who live in rural communities face barriers and challenges to accessing diabetes care (*6*). Rural populations have higher prevalence of diabetes and lower rates of participation in preventive care practices (*7*,*8*). A complex array of individual, provider, and environmental factors influence access and use of DSME by persons with diabetes who live in rural communities, including insurance, education and income, literacy, transportation, poverty, and race/ethnicity. *Anderson’s Behavioral Model for Health Services Use* describes these as predisposing and enabling factors (*8*,*9*). Challenges also include establishing and sustaining DSME programs in rural communities (*6*).

Although previous studies have described DSME use by persons with diabetes at the national level, the distribution of established DSME programs in rural communities across the United States, an enabling factor in DSME use, has not been previously described (*8*). This report analyzes DSME program data from 2016 and data from the *Atlas of Rural and Small-Town America* to describe the distribution of established DSME programs in rural counties in the United States and differences in county-level characteristics of those counties with and without a DSME program.

## Methods

### Data Sources

Data analyzed for this report originated from several data sources. This report includes DSME programs recognized by the American Diabetes Association (ADA) or accredited by the American Association of Diabetes Educators (AADE) as of July 2016 (i.e., active programs). ADA and AADE identified the addresses (i.e., physical locations) of these active programs and shared them with CDC. ADA and AADE accredit or recognize DSME programs that meet the National Standards for Diabetes Self-Management Education and Support and serve as certifying organizations for the Centers for Medicare and Medicaid Services and other third-party insurers for reimbursement. The U.S. Census Bureau’s census geocoder was used to identify published addresses and the county of each DSME program site. An internet search was conducted to identify all counties of addresses not identified by geocoding. County characteristic data originated from the U.S. Census Bureau, including the 2010 U.S. Census of Population, the American Community Survey (ACS) (2008–2012), the Small Area Income and Poverty Estimates (SAIPE), and the Small Area Health Insurance Estimates (SAIHE). The U.S. Department of Agriculture’s Economic Research Service previously compiled these county data, excluding insurance estimates, for the *2013 Atlas of Rural and Small-Town America* data set. SAIPE and SAIHE U.S. census programs use the decennial census, ACS, and numerous other administrative data sources to estimate income-related and health insurance indicators. County levels of diagnosed diabetes prevalence and incidence and number of persons with diagnosed diabetes for 2013 were previously estimated by CDC (*10*).

### Variables

This study defined nonmetropolitan counties using the rural-urban continuum code from the Economic Research Service available from the *2013 Atlas of Rural and Small-Town America* data set. This code included six nonmetropolitan categories of 1,976 urban and rural counties (62% of counties) outside of metropolitan boundaries and having no cities with ≥50,000 residents. Characteristics of these nonmetropolitan counties and populations were described and compared using several variables. The variables included number of DSME programs, DSME program density (number of DSME programs per 1,000 persons with diagnosed diabetes), total population, population density (number of persons per square mile), net migration rate, percentage foreign born, percentage of non-English–speaking households, percentage of persons aged ≥65 years, race/ethnicity percentages, poverty and unemployment rates, per capita income, percentage insured, and unadjusted and age-adjusted diabetes prevalence and incidence. 

### Data Analysis

The distribution of DSME programs in nonmetropolitan counties and differences in county characteristics between counties with and without a DSME program are described. In descriptive analyses, DSME program statuses for each of these county characteristics were compared. For comparison of continuous variables, the t-test was used for normally distributed variables, and the Wilcoxon rank-sum test was used for skewed variables. Pearson’s chi-square test for proportions was used to test associations between categorical variables.

The probability of having at least one DSME program versus no DSME program was modeled using logistic regression. The model was designed to assess the degree to which the differences between DSME program statuses can be accounted for by differences in the distribution of county characteristics. The dependency between the composite variables (i.e., variables with subgroups measured in percentages that sum to 100%) was addressed by grouping the subgroups or by using only the largest subgroups of the variable. Specifically, for the variable measuring education, the percentage of the population with less than a high school education and the percentage of the population with a high school education were combined. For the race/ethnicity variable, the smallest subgroups, Asians and Native Americans, were excluded from the multivariable analyses.

After screening all variables, variable selection was performed by fitting the model with all potential predictors and interactions terms. Variables in the model were assessed for improving the model fit using the likelihood ratio test by manual stepwise selection procedure. All variables were tested for confounding effect using the change-in-estimate approach with a 10% cut-off value. The variables in the final model were screened for multicollinearity. Loess, generalized additive model (GAM), and other graphical assessment methods were used to check for violation of linearity in the logit for the continuous variables. Model fit was assessed by Pearson’s and deviance chi-square tests, Hosmer-Lemeshow and Stukel tests, and Tjur’s statistics. Results were considered statistically significant at p<0.05. Multivariable analyses were performed in SAS 9.3 (SAS Institute, Cary, North Carolina) using proc logistic for model fitting and proc genmod for model assessment. SAS/GRAPH 9.3 (SAS Institute, Cary, North Carolina) was used to map DSME programs by county.

## Results

In 2016, a total of 1,065 DSME programs were located in 743 of the 1,976 (38%) nonmetropolitan counties or county equivalents, and 62% of nonmetropolitan counties did not have a DSME program (Figure). The total number of DSME programs for nonmetropolitan counties with at least one DSME program ranged from 1 to 8, with an average of 1.4 DSME programs. The number of DSME programs in nonmetropolitan counties of a state ranged from one in Massachusetts (with three nonmetropolitan counties) to 79 in Minnesota (with 60 nonmetropolitan counties), with an average of 22.7 DSME programs per state for nonmetropolitan counties. The proportion of nonmetropolitan counties within a state with a DSME program ranged from one of the 13 nonmetropolitan counties in Nevada to both of the nonmetropolitan counties in Hawaii and the one nonmetropolitan county in Connecticut. An average of 44.2% of nonmetropolitan counties in a state had a DSME program.

**FIGURE:**
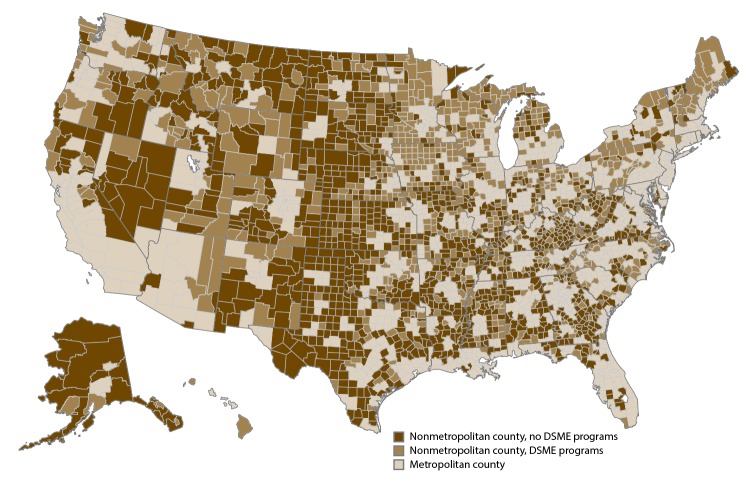
Diabetes self-management education programs in nonmetropolitan counties — United States, 2016 **Abbreviation:** DSME = diabetes self-management education. **Sources:** Addresses of active programs were obtained from the American Diabetes Association and American Association of Diabetes Educators, July 2016.

A bivariate analysis comparing county-level characteristics between nonmetropolitan counties without a DSME program and nonmetropolitan counties with at least one DSME program was conducted (Table 1). Nonmetropolitan counties with at least one DSME program had, on average, larger populations with higher population densities and net migration rates than nonmetropolitan counties without a DSME program. Nonmetropolitan counties with at least one DSME program also had a lower percentage of blacks and Hispanics, a lower percentage of non-English–speaking households, and a lower percentage of persons aged ≥65 years. Counties with at least one DSME program also were, on average, more affluent, with a higher average median household income, a lower poverty rate, a lower unemployment rate, and a lower percentage of the population with less than a high school education. In addition, a higher percentage of persons in these nonmetropolitan counties were insured. Although more persons with diagnosed diabetes lived in nonmetropolitan counties with at least one DSME program, crude and age-adjusted diabetes prevalence and incidence were lower in counties with at least one DSME program. Estimates of undiagnosed diabetes were not included in this analysis.

**TABLE 1:** Comparison of nonmetropolitan county characteristics in counties with and without a diabetes self-management education program* — United States, 2016

Characteristic	Counties with no DSME program N = 1,237 (62.47%)	Counties with at least one DSME program N = 743 (37.51%)	p value^†^
	Mean (SD)	Mean (SD)	
**Program**			
No. of DSME programs per county	0 (0)	1.4 (0.9)	—
DSME program density^§^	0 (0)	0.8 (0.8)	—
**Population**			
Population	16,188.5 (14,996.1)	35,456.7 (25,947.4)	<0.001
Population density^¶^	32.3 (72.2)	60.5 (121.9)	<0.001
Net migration rate**	-3.3 (9.1)	-0.60 (7.5)	<0.001
**Diabetes epidemiology **			
No. of persons with diabetes	1,457.7 (1,386.8)	2,941.6 (2,226.0)	<0.001
Diabetes prevalence, %	11.8 (2.6)	11.1 (2.4)	<0.001
Diabetes incidence	9.7 (2.5)	9.0 (2.4)	<0.001
Diabetes age-adjusted prevalence, %	9.8 (2.4)	9.4 (2.1)	<0.001
Diabetes age-adjusted incidence rate, per 1,000	8.8 (2.5)	8.2 (2.3)	<0.001
**Race/Ethnicity**			
White, non-Hispanic, %	77.7 (21.6)	83.8 (17.4)	<0.001
Black, %	8.8 (16.1)	5.6 (12.2)	<0.001
Hispanic, %	9.3 (15.8)	5.6 (8.9)	<0.001
Asian, %	0.5 (1.5)	0.7 (1.5)	<0.001
Native American, %	2.3 (8.4)	2.6 (9.3)	0.43
**Other demographics**			
Non–English-speaking households, %	4.1 (7.3)	2.9 (4.8)	<0.001
Foreign born, %	3.7 (5.1)	3.2 (3.3)	0.004
Persons aged ≥65 years, %	17.4 (4.2)	16.7 (3.6)	<0.001
Insured, %	83.6 (5.3)	86.8 (4.7)	<0.001
**Income **			
Median household income (thousands), $	42.4 (9.5)	45.1 (8.6)	<0.001
Per capita income of county (thousands), $	22.2 (5.01)	23.3 (4.3)	<0.001
Poverty rate, %	18.7 (7.1)	16.7 (5.9)	<0.001
Unemployment rate, %	5.8 (2.4)	5.4 (1.8)	<0.001
**Educational attainment**			
Less than high school education, aged ≥25 yrs, %	17.4 (7.4)	13.8 (5.9)	<0.001
High school education, aged ≥25 yrs, %	36.7 (6.1)	36.1 (6.4)	0.03
Some college, %	22.0 (4.2)	22.0 (3.7)	0.88
College and higher, %	16.4 (6.2)	19.1 (7.0)	<0.001

**Abbreviations:** DSME = diabetes self-management education; SD = standard deviation.

* DSME programs recognized by the American Diabetes Association and accredited by the American Association of Diabetes Education.

^†^ By two-sided t-test, Wilcoxon sum rank test, or Pearson’s chi-square test.

^§^ Number of DSME programs per 1,000 persons with diagnosed diabetes.

^¶^ Number of persons per square mile.

** Change in county population due to net migration as a percentage of the initial population, 2010–2015.

A multivariate logistic regression analysis of the association between county characteristics and the existence of at least one DSME program in a nonmetropolitan county was conducted (Table 2). After adjusting for the other factors, the odds of a nonmetropolitan county having at least one DSME program increased as the percentage insured increased (adjusted odds ratio [AOR] = 1.10, 95% confidence interval [CI] = 1.08–1.13), the percentage with a high school education or less decreased (AOR = 0.94, 95% CI = 0.93–0.96), the unemployment rate decreased (AOR = 0.84, 95% CI = 0.78–0.90), and the natural logarithm of the number of persons with diabetes increased (AOR = 3.63, 95% CI = 3.15–4.19).

**TABLE 2:** Multiple logistic regression results from analysis of associations between nonmetropolitan county characteristics and diabetes self-management education program status*

Variables	AOR^†^	95% CI
No. of persons with diabetes	3.63	3.15–4.19
Insured, %	1.10	1.08–1.13
High school education or less, aged ≥25 yrs, %	0.94	0.93–0.96
Unemployment rate, %	0.84	0.78–0.90

**Abbreviations**: AOR = adjusted odds ratio; CI = confidence interval (Wald); DSME = diabetes self-management education.

* DSME program status: no DSME program or at least one DSME program.

^†^ All p values are <0.001. The odds ratios correspond to one percentage point change in the variables measured in percentages and one unit change in the natural logarithm of the number of persons with diabetes.

## Discussion

In 2016, 62% of nonmetropolitan counties did not have a DSME program. These counties were less affluent, had more black and Hispanic persons, and had higher prevalence and incidence rates of diabetes compared with nonmetropolitan counties with at least one DSME program. The number of persons with diabetes, percentage insured, percentage with a high school education or less, and percentage unemployed was significantly associated with whether a DSME program was located in a nonmetropolitan county. These findings underscore the need to further examine individual and community-level barriers to accessing quality DSME services in nonmetropolitan, socially disadvantaged communities.

The absence of DSME programs in approximately two thirds of nonmetropolitan counties aligns with reports of challenges sustaining health care services and health professionals in rural communities (*11*). Recent national assessments of the rural workforce identified shortages of health professionals across the United States, including registered nurses, dieticians, and health educators who provide services to DSME programs (*11*). Previous attempts to expand DSME programs in clinics in rural communities have encountered challenges with recruiting health professionals required to meet the standards of DSME program recognition (*6*).

The lower percentage of the county population that is insured and employed in counties without a DSME program (compared with those with a DSME program) is also consistent with previous findings of lower health insurance coverage rates in rural communities, particularly remote rural communities (*12*). Health insurance coverage is considered a substantial expense for many persons in rural areas because the costs exceed 10% of after-tax household income (*13*). Previous studies suggest that lower insurance coverage rates at the county level in the United States contributes to underuse of diabetes preventive services by persons with a diagnosis of diabetes (*9*).

The finding of lower rates of college education in nonmetropolitan counties without a DSME program (compared with those with a DSME program) also align with the lower socioeconomic status of rural communities. In a 2010 report, the Council of Economic Advisors highlighted the persistent educational gap between urban and rural communities, with a 10%–15% difference in the likelihood of adults in rural populations attending college compared with adults in urban populations (*13*). Although the study in this report included only nonmetropolitan counties, the persons in nonmetropolitan counties without a DSME program were also less educated than those in nonmetropolitan counties with at least one DSME program. Previous national studies suggest an association between lower education levels and lower use of preventive care practices (*9*).

## Limitations

The findings in this report are subject to at least three limitations. First, DSME programs recognized by ADA or accredited by AADE in this report include only primary sites and semi-independent sites. Some sites provide DSME program services at other off-site locations not included in this study (e.g., nursing facilities, work sites, and other community settings), with a small percentage of DSME programs using telemedicine services. In addition, the wide variation in the geographic size, contiguity, or proximity of counties could influence geographic accessibility for persons with diabetes who live in counties with and without a DSME program. As a result, analysis at the county level might underestimate or overestimate geographic accessibility to DSME programs. Second, by only including ADA-recognized and AADE-accredited programs, the gaps in DSME services might be overemphasized. For example, 44 states and the District of Colombia offer licensed Stanford Diabetes Self-Management Programs. However, Medicare and many private insurance plans require that programs be recognized by ADA or accredited by AADE for reimbursement; few Stanford Diabetes Self-Management Programs have achieved recognition or accreditation (L. Kolb, AADE, personal communication, 2017). Finally, this report analyzed county-level characteristics of the general county population. The characteristics of persons with diagnosed diabetes in those counties could differ from the overall county population. Although previous studies of diabetes care services found an association between persons living in socially disadvantaged counties and lower use of diabetes education among persons with diagnosed diabetes, this report limited interpretations of findings to the county level (*9*).

## Conclusion

Monitoring the distribution of reimbursable DSME programs at the county level provides valuable insight needed to strategically address rural and other disparities in diabetes care and outcomes (*14*). These contextual-level findings of DSME program distribution, in conjunction with county-level estimates of diabetes prevalence, incidence, and other relevant data, provide the information needed to assess and address the significant gaps in the availability of DSME services. County-level data also can be used to identify opportunities to explore innovative technology such as telemedicine to deliver DSME and diabetes care in underserved rural communities with small populations and limited resources (*15*). Additional research is needed to further understand the factors influencing the geographic distribution of DSME programs in rural communities, including successful models for establishing and sustaining DSME programs in these communities.

This report found that, in 2016, few DMSE programs existed in nonmetropolitan, socially disadvantaged counties in the United States. The number of persons with diabetes, percentage insured, percentage with a high school education or less, and percentage unemployed were significantly associated with whether a DSME program was located in a nonmetropolitan county. These findings highlight the need to examine more comprehensively the barriers and challenges to accessing quality DSME programs in these communities.
